# Metabolic Profiling of Glucose-Fed Metabolically Active Resting *Zymomonas mobilis* Strains

**DOI:** 10.3390/metabo10030081

**Published:** 2020-02-26

**Authors:** Katsuya Fuchino, Uldis Kalnenieks, Reinis Rutkis, Mara Grube, Per Bruheim

**Affiliations:** 1 Department of Biotechnology and Food Science, NTNU Norwegian University of Science and Technology, 7491 Trondheim, Norway; Katsuya.Fuchino@ntnu.no; 2 Institute of Microbiology and Biotechnology, University of Latvia, LV 1004 Riga, Latvia; uldis.kalnenieks@lu.lv (U.K.); reinis.rutkis@lu.lv (R.R.); mara.grube@lu.lv (M.G.)

**Keywords:** *Zymomonas mobilis*, metabolomics, liquid chromatography-tandem mass spectrometry, Entner–Doudoroff pathway, aerobic respiration, kinetic modelling

## Abstract

*Zymomonas mobilis* is the most efficient bacterial ethanol producer and its physiology is potentially applicable to industrial-scale bioethanol production. However, compared to other industrially important microorganisms, the *Z. mobilis* metabolome and adaptation to various nutritional and genetic perturbations have been poorly characterized. For rational metabolic engineering, it is essential to understand how central metabolism and intracellular redox balance are maintained in *Z. mobilis* under various conditions. In this study, we applied quantitative mass spectrometry-based metabolomics to explore how glucose-fed non-growing *Z. mobilis* Zm6 cells metabolically adapt to change of oxygen availability. Mutants partially impaired in ethanol synthesis (Zm6 *adhB*) or oxidative stress response (Zm6 *cat*) were also examined. Distinct patterns of adaptation of central metabolite pools due to the change in cultivation condition and between the mutants and Zm6 reference strain were observed. Decreased NADH/NAD ratio under aerobic incubation corresponded to higher concentrations of the phosphorylated glycolytic intermediates, in accordance with predictions of the kinetic model of Entner–Doudoroff pathway. The effects on the metabolite pools of aerobic to anaerobic transition were similar in the mutants, yet less pronounced. The present data on metabolic plasticity of non-growing *Z. mobilis* cells will facilitate the further metabolic engineering of the respective strains and their application as biocatalysts.

## 1. Introduction

Over recent decades, there has been an ever-growing interest in the production of renewable biofuels. Along with yeasts, the alphaproteobacterium *Zymomonas mobilis* has been attracting significant attention for usage in large-scale biofuels bioprocessing [1]. *Z. mobilis* possesses several desirable features for biofuels production, such as high specific rate of sugar uptake, presence of active pyruvate carboxylase, tolerance to high concentration of ethanol (11–16% v/v) and broad range of pH (3.5–7.5), low biomass production, and relatively small genome size [1,2,3]. *Z. mobilis* utilizes Entner–Doudoroff (ED) pathway and can produce ethanol as a final catabolic product at nearly theoretical yield of 0.51 g EtOH/g glucose [3,4]. This bacterium generates only 1 molecule of ATP per catabolizing 1 molecule of glucose, and it has also been shown that its Tricarboxylic acid cycle (TCA) is truncated and Pentose phosphate pathway (PPP) is incomplete [5].

Another distinctive physiological characteristics of *Z. mobilis* is its high capacity of aerobic respiration, proceeding almost without concomitant energy production [3,6,7,8]. The primary electron donor in *Z. mobilis* electron transport chain is NADH [9,10], therefore aerobic respiration results in acetaldehyde production by withdrawal of NADH from ethanol synthesis [3,11]. Yet the full scale of physiological consequences of aeration in *Z. mobilis* remains to be elucidated. For example, it was lately found that anaerobic to aerobic transition significantly influences mevalonate pathway in growing *Z. mobilis* cells [12]. Also, a recent phospho-proteome analysis has revealed that several ED enzymes in *Z. mobilis* cells are phosphorylated during aerobic, but not anaerobic condition [13], pointing to some still unknown modes of regulation within the ED pathway.

Although the high rate of fermentative catabolism and the uncanonical bioenergetics of its aerobic respiration position this bacterium as an efficient producer of catabolic end-products, yet, relative to other industrial workhorses like *Escherichia coli* and *Saccharomyces cerevisiae*, the metabolic plasticity of this organism has been poorly characterized. Furthermore, introducing heterologous expression has expanded the potential of *Z. mobilis*, for example enabling consumption of xylose and other substrates for biofuel production [14,15], but at the same time, metabolic engineering may result in unpredicted alteration of central metabolic fluxes and also influence the intracellular redox balance. It is important therefore to deepen our basic understanding of *Z. mobilis* central metabolism, redox homeostasis and its relation to the operation of the ED catabolic pathway.

A study by Yang et al. [16] was the first to characterise growing *Z. mobilis* cells under aerobic and anaerobic situations by metabolomics and transcriptomics. Concerning the intracellular metabolome, these authors focussed on stationary phase cells with near-zero glucose concentration left in their growth media, and used GC-MS metabolite profiling with limited coverage of central metabolite pools. Another recent work [12] monitored the metabolome of exponentially growing cultures during anaerobic to aerobic transition. Kalnenieks et al. [17] reported metabolomes of aerobic batch cultures of several *Z. mobilis* mutants. These studies revealed dynamic shift of the metabolome and some of its consequences in growing *Z. mobilis* cells.

However, to the best of our knowledge there is no published work on metabolomics of metabolically active non-growing *Z. mobilis* cells. Resting biocatalyst state with high biomass recycling rates is frequently applied as an industrial bioprocess mode of operation to maximize product yields and minimize carbon loss due to biomass synthesis [18,19]. For a bacterium like *Z. mobilis*, incorporating less than 5% of substrate carbon into its biomass [5] and performing catabolism at high rate, metabolism of resting cells represents special biotechnological interest. Exploiting of the resting cell state of *Z. mobilis* has been attempted since seventies [20], much in the context of immobilized cell preparations [21,22,23] and thus, profiling the metabolome of resting *Z. mobilis* cells is of practical importance. Furthermore, application of metabolically active non-growing cells is not restricted to ethanol synthesis, but potentially can serve also for the synthesis of aerobic catabolic products, like acetaldehyde and several other compounds [24].

In the present study, we therefore analysed metabolic profiles of *Zymomonas mobilis* subsp. ATCC 29191 (Zm6) and its mutant derivatives, catalase and alcohol dehydrogenase knock-out strains with altered aerobic physiology at metabolically active non-growing (resting) state under aerobic and anaerobic condition. Such a comparative metabolomic analysis exposed the scale of catabolic plasticity of *Z. mobilis* without interfering with pathways related to cellular growth.

## 2. Results and Discussion

### 2.1. Shutdown of Cellular Growth Significantly Alters Zymomonas Mobilis Metabolome, Yet Partly Retains Its High Catabolic Rate

**Figure 1:**
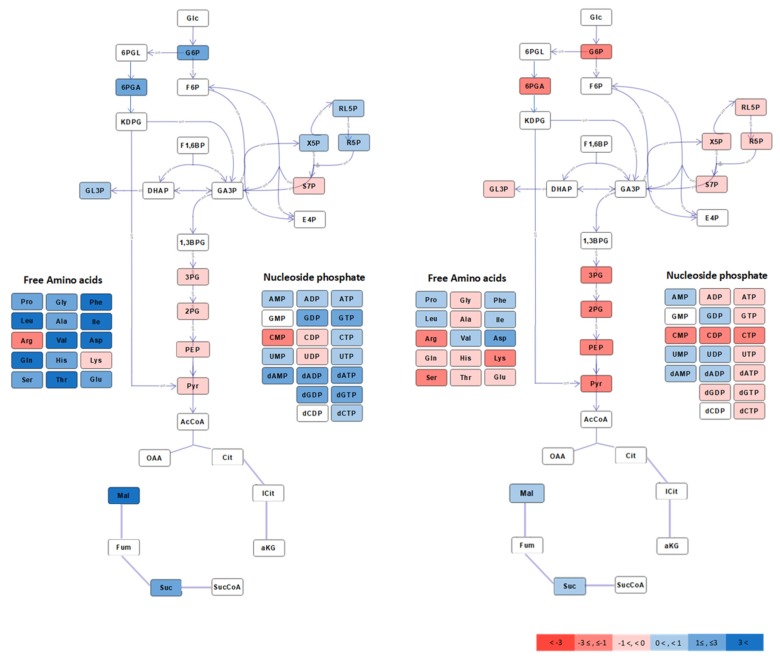
A schematic overview of central carbon metabolism in *Z. mobilis*, coloured by log2 heatmap of intracellular metabolites concentration in aerobically growing to aerobically resting Zm6 cells (left) and anaerobic to aerobic resting Zm6 cells (right).

First, a comparison of growing cells and glucose-fed resting cells was performed to investigate how exclusion of cellular growth impacts the central carbon metabolite pools of Zm6. The result from target quantitative mass spectrometry-based metabolite profiling is presented in log2 ratios of resting vs growing Zm6 cells (Figure 1, left). The analysis revealed quite distinct patterns with larger amino acid and nucleotide pools in growing than in resting cells (Figure 1, left). Glucose consumption rate in aerobically growing Zm6 cells (30.22 ± 0.41 mmol/cell dry weight g/h) was about 2.5 times higher than in resting cells (Table 1). Notably, this value exceeds almost three times the aerobic glucose consumption rate recently reported for aerobically growing *E. coli* strain, engineered for high glucose throughput [25]. The rate of glucose consumption in non-growing *Z. mobilis* is thus closely comparable to that seen in growing high glucose throughput strain of *E. coli*. These results are in agreement with the pattern of ‘uncoupled growth’, earlier observed in *Z. mobilis* [7]. The resting state of *Z. mobilis* cell is metabolically active, which is important for industrially relevant bioprocess setting. In addition, we envision that this system could be exploited for highlighting the metabolic plasticity of *Z. mobilis* under different conditions.

For interpretation of metabolite pool data, it is critical to realize that there is no direct proportionality between pool sizes and fluxes. Quite the opposite can take place in some situations, e.g., in a linear pathway where accumulation of the end-product feedback inhibits the first reaction in the pathway [26]. Nevertheless, the increased amino acid and nucleotide pools in growing cells were expected, since in growing cells all biosynthetic pathways were active, while in resting state they were down-regulated. Shutting down the metabolic fluxes to nucleotides and amino acid biosynthesis also led to redistribution among central carbon metabolite pools. The upper ED-pathway metabolites G6P and 6PGA were down-regulated while the lower ED pathway metabolites PEP, 3PG-2PG and Pyr were significantly increased in resting cells (Figure 1, left). This was not due to altered redox-balance, since NADH/NAD ratio did not dramatically change after stopping growth (Table 2), nor was the adenylate energy charge significantly different in each case (Table S1).

### 2.2. Aerobic to Anaerobic Transition Upregulates NADH/NAD, yet Downregulates ED Pathway Metabolite Pools

Next, we compared metabolite pool profiles of non-growing, steady-state Zm6 cells under aerobic and anaerobic condition. The resting cell cultures were first incubated under aerobic condition, and then switched to anaerobic condition by flushing nitrogen gas instead of air. Samples were taken after a steady rate of glucose consumption had been established both under aerobic and anaerobic condition, avoiding sampling during the transition state (see Methods). Interestingly, all ED pathway metabolite pools were downregulated under anaerobic condition (Figure 1, right), although the specific glucose consumption rate almost doubled after switching to nitrogen gassing (Table 1).

**Figure 2:**
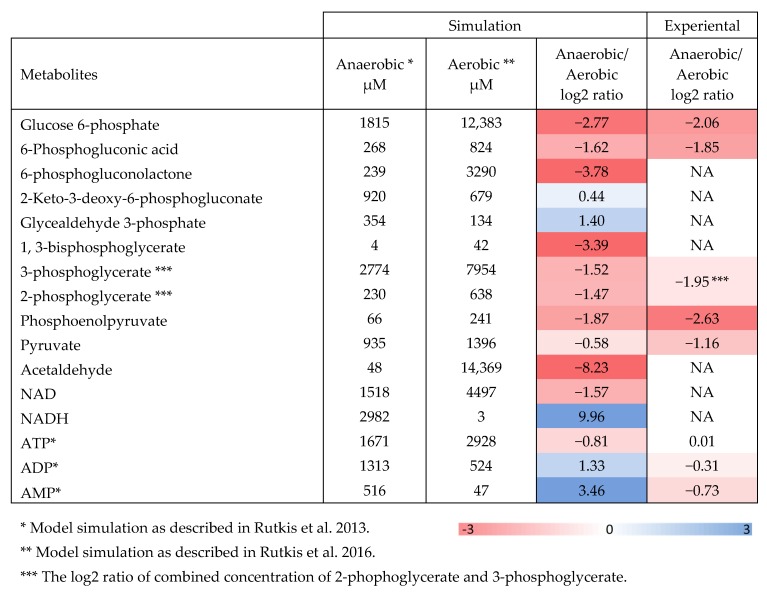
In silico simulation of ED intermediate pools in resting *Z. mobilis* cells and comparison to the experimental data. The simulation was conducted employing ED pathway kinetic model in non-growing *Z. mobilis* as described in [8,27].

To see if this could be anticipated from the known kinetic parameters of the ED pathway, we ran simulations of the aerobic to anaerobic transition (Figure 2) using the ED pathway kinetic model for non-growing *Z. mobilis* [8,27]. Our simulations supported higher metabolite pools (compare the data on glucose-6-phosphate, 6-phosphogluconate and the ED intermediates downstream glyceraldehyde-3-phosphate; Figure 2) and lower NADH/NAD ratio under aerobic incubation, like found in the present experiments. Elevation of pools downstream glyceraldehyde-3-phosphate during the anaerobic to aerobic transition is supported also by data of Martien et al. [12] on growing cells. Besides, the model suggests that lowering of NADH/NAD ratio should lead to higher rate of glycolysis via the *Z. mobilis* ED pathway, apparently because faster glycolysis requires more NAD for metabolizing sugar [28], and that the glyceraldehyde-3-phosphate dehydrogenase (GAPDH) might be the reaction, initially responding to the drop of NADH/NAD. Interestingly, apart from stimulation of the glycolytic rate, several recent studies indicate that low NADH/NAD is particularly beneficial under various stress conditions, including saline and acetic acid stress [28,29,30,31].

Under anaerobic conditions, the observed specific rate of glucose consumption in Zm6 was around 20 mmoles/g dry weight hr, reasonably close to the model-generated value of 27 mmoles/g dry weight hr [8]. However, in accordance with the lowered NADH/NAD ratio (but in contrast to the present experimental results), the model predicted higher glucose uptake rate under aerobic conditions. We speculate that such a discrepancy between the model simulations and the present experiments could at least in part be caused by the inhibitory effect of acetaldehyde. The model does not take into account possible inhibitory action of the accumulated acetaldehyde, therefore, the simulation results have been previously verified experimentally, using vigorous gassing of air (2.5 v/v per min) through the culture to remove most of acetaldehyde [8]. Under such vigorous gassing, an aerobic increase of glycolytic rate actually did take place. In the present work at 0.8 v/v per min aeration acetaldehyde was not thoroughly gassed out (like in most of aerobic growth experiments with *Z. mobilis*), so its inhibitory effect could largely contribute to the slowdown of aerobic glycolysis. At the end of aerobic incubation its concentration in cell suspensions of Zm6 and Zm6 *adhB* had reached 0.3 g L^−1^, while in Zm6 *cat* it was close to 0.4 g L^−1^ (not shown), which was in the range of acetaldehyde concentrations showing a distinct inhibitory effect [11]. Remarkably however, in both experimental setups and in model simulation the aerobic pools of ED metabolites were higher than the anaerobic ones, independently of the difference of glucose consumption rates in each case. From this we conclude that there is no relation between the net glycolytic flux rate and the concentration of phosphorylated intermediates in *Z. mobilis* during the aerobic to anaerobic transition.

As expected, the ethanol yield upon transition to anaerobic condition also increased, from 0.22 to 0.39 (ethanol (g)/glucose (g)). Apparently, the absence of oxygen as the terminal electron acceptor directed more reducing equivalents to reduction of acetaldehyde by alcohol dehydronase. However, Zm6 cells still maintained a significantly higher NADH/NAD ratio under anaerobic conditions (Table 2). Only minor adjustments were observed among amino acids and nucleotides during the shift. Even though ATP was slightly downregulated, the anaerobic Zm6 cells kept almost the same energy charge as that of aerobic cells (Table S1). That is not surprising since the main ATP generating mechanism in both aerobic and anaerobic Zm6 cells is substrate-level phosphorylation [3].

### 2.3. Metabolite Profiling of Mutants With Partially Impaired Ethanol Synthesis (Zm6 adhB) or Enhanced Respiratory Capacity (Zm6 cat) Exhibit Common Trends

Next, we performed the same metabolite profiling approach on resting Zm6 *cat* and Zm6 *adhB* mutants. The Zm6 *cat* mutant was included in this study due to its interesting phenotype, an elevated aerobic respiration capacity [32], and accordingly, an improved capacity of acetaldehyde synthesis [31]. This strain was shown to be sensitive to hydrogen peroxide due to the lack of catalase activity [32], but the mutation did not cause growth deficiency under aerobic condition, suggesting that the mutant was still able to cope with general oxidative stress [17]. The mechanism by which the mutant upregulates oxygen consumption is not understood, but it is likely that its redox dynamics is altered. In *Z. mobilis*, there are two alcohol dehydrogenase enzymes (AdhI encoded by *adhA* and AdhII encoded by *adhB*). The Zm6 *adhB* strain was chosen for metabolomic study because of its altered redox dynamics [33,34], and also its potential use for industrial acetaldehyde production due to lower ethanol-synthesising activity [17].

**Figure 3:**
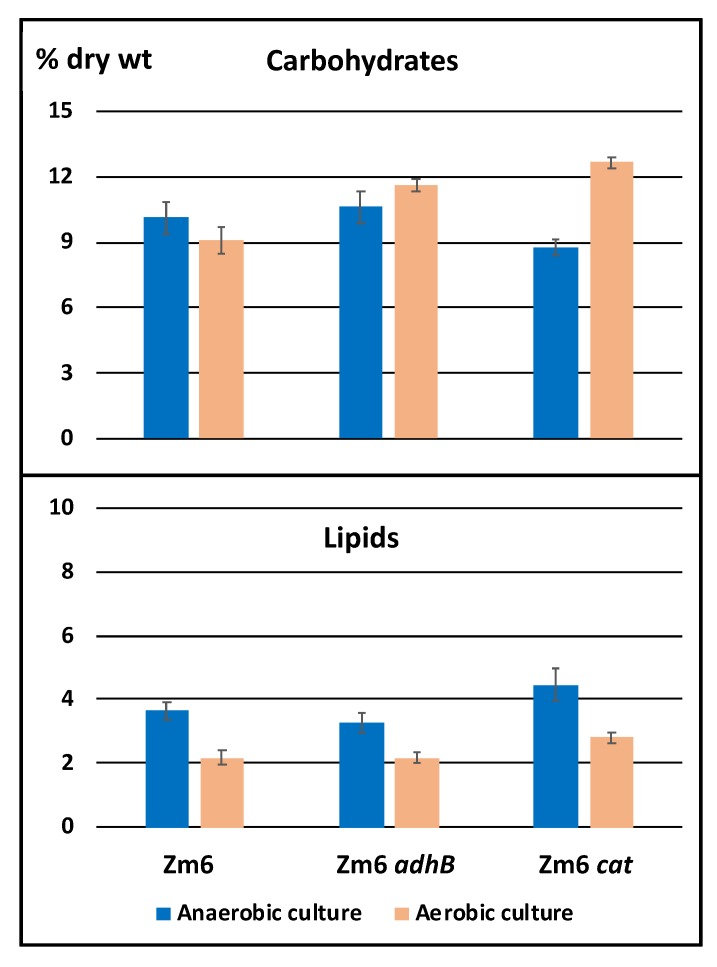
Total carbohydrate and lipid content (% of dry weight) in the stationary phase cells of Zm6, Zm6 *adhB* and Zm6 *cat* strains, grown under anaerobic or aerobic conditions.

The effect of aeration upon the cellular content of protein, nucleic acids, carbohydrates and lipids (in percent of cell dry weight) in all three strains was estimated from the Fourier-transform infrared spectra (FTIR) of the biomass samples. This study was undertaken as a complimentary investigation to assess if major changes in biomass composition in mutant strains could be direct explanations to potential differences in metabolite profiles. FTIR is a rapid method, particularly suited for monitoring the relative change of the cellular content of each macromolecular component under varying conditions [35]. In the present study the infrared spectra revealed a pattern of macromolecular composition of stationary phase cells, which was largely similar to previously reported data, obtained by alternative methods [36,37]. Protein varied in the range between 59 and 64 % of cell dry weight, while nucleic acids constituted 14.5–17 % (not shown). Both these components showed no significant variability between the strains or aeration conditions. However, in all three strains the lipid content was lower under aerobic condition; a characteristics indicative for oxidative stress condition [38]. In addition, in Zm6 *cat* strain aerobically cultivated cells showed an increase in carbohydrate content (Figure 3).

The glucose consumption rate during both aerobic and anaerobic resting state was similar to Zm6 reference in the Zm6 *cat* mutant (Table 1). The ethanol yields in Zm6 *cat* were lower, especially under aerobic conditions, supporting the phenotype of Zm6 *cat* mutant with higher respiratory rate and accumulation of acetaldehyde [17]. Interestingly, the NADH/ NAD ratio was similar to that of Zm6 under aerobic conditions, whereas its increase was not observed in Zm6 *cat* under transition to anaerobic conditions; rather a slight decrease took place (Table 2). In general, deletion of *cat* locus had a high impact on the central carbon metabolism under aerobic condition (Table 3a, see the left column, Zm6 *cat*/Zm6), causing aerobic downregulation of most ED-pathway intermediate metabolites. This observation further supports the absence of correlation between the net glycolytic flux rate and the concentration of phosphorylated intermediates, since the ED pathway flux in Zm6 *cat* strain is very similar to that in Zm6.

The Zm6 *adhB* strain exhibited similar glucose consumption rates to Zm6 in aerobic condition, but significantly lower under anaerobic situation (Table 1). The ethanol yield was lower in both conditions, probably due to accumulation of acetaldehyde. Ethanol in the Zm6 *adhB* mutant is produced only by AdhI and that enables maintenance of the glucose uptake and intracellular conversion to ethanol at about 75% of the wild type reference. Introduction of a flux limitation at the last step of ethanol biosynthesis pathway had consequences for the upstream metabolite pools. In the aerobic situation several ED pathway metabolites were down-regulated, while most of them being upregulated in the anaerobic situation, relative to the Zm6 reference (Table 3b, see the left column). The NADH/NAD ratio in the Zm6 *adhB* mutant tended to increase under anaerobic condition, exhibiting the same pattern as in Zm6, yet the change was much smaller. The dynamics of the ED intermediates around the pyruvate node differed most between Zm6 *adhB* and Zm6.

Taken together, we found that relative to Zm6, deletion of *cat* and *adhB* locus elicited similar global trends in the central carbon metabolites, both under aerobic and anaerobic conditions (compare the left and middle columns in Table 3a and b). These two mutations both led to narrower intervals of change of NADH/NAD ratio and of phosphorylated intermediate concentrations, than seen in the parental strain upon the aerobic to anaerobic transition. In all cases, the lower NADH/ NAD ratio corresponded to higher concentrations of the phosphorylated glycolytic intermediates, in accordance with the model predictions [8]. Furthermore, the magnitude of the NADH/NAD ratio is expected to influence acetaldehyde production, although via different mechanisms (increase of respiratory activity in *cat* vs. decrease of alcohol dehydrogenase activity in Zm6 *adhB*). Apparently, the aerobic overproduction of the inhibitory metabolite acetaldehyde in both these strains (in addition to the effect of NADH/NAD ratio per se) largely determined the observed metabolite pool balance around the pyruvate node and also upstream the ED pathway.

In conclusion, our results exemplify that the control of central metabolic fluxes is a complex phenomenon and predicting all consequences of the strain metabolic engineering at sufficient precision is still challenging. This unpredictability is particularly pronounced when toxic volatile compounds are produced and excreted in the medium during cultivation, like acetaldehyde in the case of *Z. mobilis*. Nevertheless, here at a semi-quantitative level the predictions of the kinetic model of the Entner-Doudoroff pathway proved to comply with the observed metabolome dynamics during transition between different modes of aeration, at least correctly simulating the directionality of change of metabolite concentrations. Acquiring more reference metabolome data on *Z. mobilis* catabolism is mandatory for improvement of the present E-D pathway kinetic model, especially with regards to simulations of *Zymomonas* aerobic catabolism. An improved kinetic model would allow more rigorous quantitative analysis of the flux, including metabolic control analysis (MCA) for highlighting the metabolic bottlenecks as the potential targets of genetic engineering. This type of interaction between metabolomics and model building would result in a better kinetic model. That in its turn would enable design of novel, perhaps counterintuitive, metabolic engineering strategies for *Z. mobilis* central metabolism, including those, directed towards use of respiratory mutants as biocatalysts for broadening of the aerobic product spectrum.

## 3. Materials and Methods

*Z. mobilis* ATCC 29191 (Zm6) was cultivated in 3 litre fermentors (Eppendorf New Brunswick 115 series bioreactor) with 1.2 litre operating volume. Cells were grown at 30 °C in a complex growth medium containing glucose (20 g/L), yeast extract (5 g/L), NH_4_SO_4_ (1 g/L), KH_2_PO_4_ (1 g/L), MgSO_4_ (0.5 g/L), as described previously [39]. The pH in the medium was adjusted to 5.5 by NaOH during all fermentation. Atmospheric air flow was set at 1.0 L/min, and off-gas from fermenters was monitored by DASGIP^®^ gas analyser (Eppendorf Bioprocess Center Europe, Jülich, Germany). Cultivation for resting cells was performed as follows. Cells were grown under fully controlled aerobic condition. The fully-grown cells that reached stationary phase were collected by centrifuge (10 min, 5000 rpm), washed twice in salt solution containing KH_2_PO_4_ (1 g/L) and MgSO_4_ (0.5 g/L), resuspended in the salt solution supplemented with 10 g/L glucose. The resting culture were incubated aerobically for 2.5 h. After that, anaerobic incubation was started by gassing with nitrogen at 1.0 L/min. Parental strain Zm6, the Zm6 *cat* [32], and the Zm6 *adhB* mutant lacking alcohol dehydrogenese [33] were chosen for resting cell experiments.

Sampling was performed at 75th and 120th minute after aerobic resting cultivation had started, and at 45th and 90th minute after switching to anaerobic condition. These sampling time points corresponded to catabolic steady state, as judged from glucose consumption rate. Quenching of samples were performed as previously described [40]. Cells were filtered through Polyethersulfone membrane disc filters (Pall), washed by cold salt solution (MgSO_4_ 0.5 g/L and KH_2_PO_4_ 1 g/L) and cold milli-Q water. Filtered samples were then quenched by ACN and H_2_O mixture (55% and 45%), and snap-freezed by liquid nitrogen. Collected cells were disrupted by 3 cycles of freezing and thawing, and lyophilized for storage at −80 °C. Prior to the analysis, the freeze-dried samples were resuspended in MS grade H_2_O (VWR) and spin-filtered with low protein binding filter (VWR).

The measurement of phosphorylated sugars and nucleotides were performed using capillary ion chromatography [40,41] coupled to tandem mass spectrometry Xevo TQ-XS Triple Quadrupole Mass Spectrometry (Waters, Milford, MA, USA). Free amino acids and Organic acids were derivatized by Edman’s reagent [42] and O-Benzylhydroxylamine [43], respectively. Derivatized samples were analysed by ACQUITY I-Class UPLC (Waters) coupled to TQ-XS. Software MassLynx (Waters) was used for data acquisition, and its application TargetLynx (Waters) was used for processing the data. Metabolite concentrations were average of two time points for each condition and normalized according to dry weight and optical density at 600 nm (OD) for a comparative analysis.

The kinetic model of *Z. mobilis* E-D pathway used in this study has been described and validated previously [8,27]. Kinetic modeling was carried out using COPASI software. The model simulations in the present work were intended to reproduce the directionality of the observed effects, without attempts to obtain quantitative precision.

For FTIR spectroscopy, bacterial biomass was washed with distilled water in triple to remove the growth medium. FTIR spectra of bacterial biomass were recorded using a high throughput screening extension (HTS-XT) coupled to a Vertex 70 spectrometer (Bruker Optik GmbH, Ettlingen, Germany) equipped with a globular mid-IR source and a DTGS detector. 5–10 µL aliquots of bacterial cell suspension in water were pipetted on a 384 well silicon microplate in three replicates and dried at T < 50 °C. Absorption spectra were collected over the wavenumber range of 4000–600 cm^−1^ with a resolution of 4 cm^−1^, 64 scans. Baseline was corrected using the rubber band method, and CO_2_ bands were excluded. For data analyses spectra in the absorption range of 0.25–0.80 were used (the range in which the concentration of a component is proportional to the intensity of its absorption band, according to the Lambert–Bouger–Beer law). Data were processed using OPUS 6.5 software. The content of carbohydrates, nucleic acids, proteins and lipids in biomass was calculated as in Grube et al. [35].

Glucose and ethanol concentrations in the spent media during resting incubation was measured using Waters 2695e Alliance HPLC (Waters). Hi-plex column (300 × 7.7 mm, Agilent, Santa Clara, CA, USA) was used at column temperature 45 °C, with 0.05 M sulfuric acid as mobile phase at a flow rate of 0.8 mL/min. The concentration in samples was calculated from a linear external standard curve. Acetaldehyde concentration was monitored enzymatically using ‘Megazyme’ analytical kit, following manufacturer’s instructions.

NADH/NAD measurement by an enzymatic assay was performed according to the instruction supplied by the manufacturer (Sigma, Saint Louis, MI, USA). Cells were collected and spin down by centrifuge at the time of samplings. Pelleted cells were then snap-frozen by liquid nitrogen and stored at −80 °C until the samples were used for measurements. Upon the analysis, cells were thawed on ice and resuspended in the buffer supplied from the kit. Absorbance at 450 nm was measured every 15 min using 96 well-plates in plate-reader spark 20M (Tecan, Männedorf, Switzerland).

## Figures and Tables

**Table 1:** Steady state glucose consumption rate (glucose mmol/cell dry weight g/h) and ethanol yield (g) per glucose (g) during resting cultivations. Each value is a mean of three technical replicates with a standard deviation.

Strain	Glucose mmol/CDW g/h		Y ethanol (g)/glucose (g)	
	Aerobic	Anaerobic	Aerobic	Anaerobic
Zm6	12.13 ± 1.50	20.15 ± 0.89	0.22 ± 0.02	0.39 ± 0.02
Zm6 *cat*	11.27 ± 0.36	19.45 ± 0.15	0.18 ± 0.02	0.32 ± 0.01
Zm6 *adhB*	12.5 ± 1.92	15.6 ± 0.53	0.18 ± 0.03	0.33 ± 0.04

**Table 2:** NADH/NAD ratio in each *Z. mobilis* strain at steady state during resting cultivation. NADH/NAD ratio is an average of 4 technical replicates. RSD = relative standard deviation.

Strains and Conditions		NADH/NAD	RSD (%)
Zm6 resting	Aerobic	0.51	38.11
	Anaerobic	0.99	30.38
Zm6 *cat* resting	Aerobic	0.57	15.1
	Anaerobic	0.46	20.8
Zm6 *adhB* resting	Aerobic	0.61	16.35
	Anaerobic	0.74	1.97
Zm6 growing	Aerobic	0.58	4.8

**Table 3:** **a** (left) and **b** (right). log2 heatmap of metabolites concentration in each resting strain versus resting Zm6 under aerobic (left column), anaerobic (middle column), and anaerobic vs aerobic in each resting strain (right column). Abbreviation of metabolites are as in Table S2. Colour gradient; scale of log2 value.

	Zm6 *cat*/ Zm6	Zm6 *cat*/ Zm6	Zm6 *cat*		Zm6 *adh*/ Zm6	Zm6 *adh*/ Zm6	Zm6 *adh*
	Aerobic	Anaerobic	Anaerobic /Aerobic		Aerobic	Anaerobic	Anaerobic /Aerobic
G6P	−2.16	0.69	0.79	G6P	−1.01	0.73	−0.32
6PGA	1.20	2.77	−0.28	6PGA	1.17	2.23	−0.79
P5P	1.57	1.91	0.25	P5P	0.94	1.98	0.94
Sedo-7p	2.05	0.81	−1.49	Sedo-7p	0.59	0.05	−0.78
Gly-3p	0.98	1.85	0.52	Gly-3p	0.68	1.96	0.94
3PG-2PG	−1.04	0.88	−0.04	3PG-2PG	−1.62	0.91	0.58
PEP	−1.98	0.22	−0.43	PEP	−2.08	0.60	0.05
Pyr	−2.20	−0.80	0.24	Pyr	−1.80	−0.40	0.24
Suc	0.27	1.52	1.38	Suc	0.60	0.66	0.18
Mal	0.93	2.07	1.42	Mal	−1.12	−0.13	1.26
AMP	0.65	−0.09	−0.73	AMP	0.13	−0.60	−0.72
ADP	0.40	0.29	−0.42	ADP	0.17	0.02	−0.46
ATP	0.04	0.53	−0.24	ATP	0.55	0.70	−0.59
GDP	0.45	0.29	0.11	GDP	0.21	−0.01	0.05
GTP	0.21	0.53	0.20	GTP	0.24	0.43	0.06
CMP	−0.18	1.63	0.19	CMP	−0.24	−0.28	−1.66
CDP	0.20	0.57	−0.63	CDP	−0.44	−0.59	−1.16
CTP	0.68	1.12	−0.71	CTP	0.63	0.88	−0.91
UMP	0.09	0.41	0.53	UMP	0.05	−0.22	−0.06
UDP	0.21	0.12	0.05	UDP	−0.08	−0.47	−0.25
UTP	0.47	0.54	−0.12	UTP	0.34	0.41	−0.12
dAMP	0.85	0.56	0.05	dAMP	−0.91	−1.08	0.17
dADP	0.63	1.27	0.82	dADP	−1.18	−1.28	0.08
dATP	−0.11	1.37	1.22	dATP	−0.92	−0.68	−0.02
dGDP	−0.09	−1.86	−2.01	dGDP	0.99	−0.49	−1.72
dGTP	−0.15	1.50	0.74	dGTP	0.30	−0.53	−1.74
dUMP	1.22	0.00	−0.81	dUMP	1.22	0.32	−0.49
dUTP	0.83	0.19	0.00	dUTP	1.22	0.10	−0.49
dCTP	0.33	0.66	−0.45	dCTP	0.40	0.08	−1.10
Gly	−0.12	0.76	0.80	Gly	−0.66	−0.48	0.12
Ala	0.52	0.35	−0.22	Ala	0.88	−0.09	−1.01
Ser	0.25	0.76	−0.65	Ser	0.00	0.69	−0.47
Pro	0.57	1.21	0.68	Pro	1.66	1.25	−0.37
Val	0.12	0.27	0.70	Val	0.27	−0.22	0.06
Thr	0.49	0.58	0.00	Thr	0.34	0.23	−0.21
Ile	0.00	0.02	0.55	Ile	0.38	−0.05	0.11
Leu	−0.04	0.10	0.85	Leu	0.38	−0.03	0.31
Asp	1.14	0.83	0.86	Asp	1.92	0.95	0.19
Gln	1.65	2.12	−0.29	Gln	1.22	0.94	−1.03
Glu	0.26	0.71	−0.13	Glu	−0.11	−0.12	−0.58
His	0.17	0.32	0.07	His	−0.20	0.15	0.26
Phe	−1.11	−0.82	0.74	Phe	−1.25	−1.67	0.03
Arg	−0.43	0.71	−0.68	Arg	−1.11	−0.04	−0.76
Lys	−0.41	0.55	−0.38	Lys	−1.36	−0.59	−0.56
				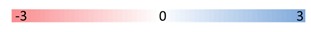			

## Supplementary Materials

The following are available online at https://www.mdpi.com/2218-1989/10/3/81/s1, Table S1: A table of concentrations of metabolites analysed in this study, Table S2: List of abbreviation of metabolites.
