# Supplementary material for: Metabolic Profiling of Glucose-Fed Metabolically Active Resting Zymomonas mobilis Strains
Source: Metabolites. 2020 Feb 26;10(3):81. doi: 10.3390/metabo10030081 (PMC7142471; doi:10.3390/metabo10030081)
Supplement: Supplementary file 1 [file metabolites-10-00081-s001.zip › Table S1.pdf]

Table S1

		resting						growing
		aerobic			anaerobic			aerobic
	Metabolites	Zm6	Zm6 *cat*	Zm6 *adhB*	Zm6	Zm6 *cat*	Zm6 *adhB*	Zm6
central carbon metabolites	G6P	498.0	111.7	247.7	119.8	193.3	198.9	1281.4
	6PGA	497.7	1141.2	1121.2	138.3	940.8	647.9	1120.5
	P5P	144.5	429.7	277.9	135.6	510,00	534.1	177.1
	Sedo-7p	185.2	768.7	278.1	156.8	274.6	161.9	166.2
	Glycerol 3p	110.2	217,00	176.2	86.4	311.5	337.1	210.4
	3PG-2PG	773.4	377.2	251,00	199.4	367.3	374.1	575.7
	PEP	73.8	18.7	17.4	11.9	13.9	18.0	45.7
	Pyr	2037.5	442.6	586.9	912.7	522.5	691.2	1888.0
	Suc	49.7	59.7	75.2	53.9	155.0	85.1	327.8
	Malate	1.7	3.2	0.8	2.0	8.5	1.9	21.0
nucleotides	AMP	860.6	1350.5	938.8	866.1	813.5	571.1	990.3
	ADP	1875.0	2477.2	2111.1	1510.1	1847.9	1533.2	2706.5
	ATP	3633.7	3731.0	5318.3	2184.0	3149.9	3536.7	6033.9
	GDP	455.4	621.0	525.2	547.8	671.1	542.8	1049.8
	GTP	1771.5	2048.5	2091.8	1627.7	2352.7	2186.2	5165.4
	CMP	1332.6	1179.8	1130.6	435.5	1344.0	357.8	634.1
	CDP	918.1	1057.9	678.8	459.8	683.2	304.5	872.6
	CTP	1192.7	1913.2	1850.3	536.4	1168.4	986.2	2239.6
	UMP	2664.5	2836.9	2753.5	3078.4	4102.8	2642.4	3457.4
	UDP	577.9	667.3	547.1	636.1	692.4	460.2	416.8
	UTP	1973.6	2724.3	2502.8	1727.3	2508.7	2302.3	2546.4
deoxy nucleotides	dAMP	1.5	2.7	0.8	1.9	2.8	0.9	3.1
	dADP	7.5	11.6	3.3	8.5	20.5	3.5	18.4
	dATP	14.0	13.0	7.4	11.7	30.2	7.3	37.6
	dGDP	22.3	20.9	44.4	18.9	5.2	13.5	50.0
	dGTP	32.7	29.4	40.2	17.3	49.0	12.0	143.7
	dUMP	0.3	0.7	0.7	0.4	0.4	0.5	0.9
	dUTP	0.9	1.6	2.1	1.4	1.6	1.5	2.4
	dCTP	12.5	15.7	16.5	7.3	11.5	7.7	12.8
amino acids	Glycine	423.7	391.2	267.9	403.7	682.1	290.3	1655.1
	Alanine	720.9	1032.2	1322.9	696.9	888.0	656.8	4833.0
	Ser	267.8	319.2	267.9	119.9	203.3	193.5	1875.3
	Pro	166.6	247.1	527.5	171.9	396.4	407.5	579.0
	Val	94.6	103,00	114.4	138.6	167.3	119.1	2775.2
	Thr	44.0	61.8	55.8	41.3	61.8	48.4	1762.0
	Ile	38.6	38.6	50.2	56.0	56.6	54.0	1151.6
	Leu	147.9	144.1	192.6	242.5	260.0	238.2	1969.7
	Asp	135.9	298.6	513.5	303.8	540.5	586.1	1145.3
	Gln	36.0	113.3	83.7	21.3	92.7	40.9	7344.0
	Glu	1660.3	1989.7	1535.0	1112.6	1822.4	1027.1	7482.4
	His	48.0	54.1	41.9	45.3	56.6	50.2	264.3
	Phe	99.9	46.3	41.9	135.9	77.2	42.8	918.8

	Arg	4787.7	3547.0	2218.8	1349.8	2211.1	1313.6	2139.6
	Lys	3239.3	2440.2	1258.7	1285.9	1876.4	852.2	2089.3
	Energy charge	0.72	0.66	0.76	0.64	0.70	0.76	0.76
