# Supplementary material for: Metabolic Profiling of Glucose-Fed Metabolically Active Resting Zymomonas mobilis Strains
Source: Metabolites. 2020 Feb 26;10(3):81. doi: 10.3390/metabo10030081 (PMC7142471; doi:10.3390/metabo10030081)
Supplement: Supplementary file 1 [file metabolites-10-00081-s001.zip › Table S2.pdf]

Table S2. List of abbrevation of metabolites

List of abbreviations of metabolites.	
G6P	Glucose 6-phosphate
6PGA	6-phosphogluconate
P5P	Ribulose 5-phosphate, Ribose 5-phosphate, Xylulose 5-phosphate
Sedo-7p	Sedoheptulose 7-phosphate
Gly-3p	Glycerol 3-phosphate
3PG-2PG	2-Phosphoglycerate, 3-Phosphoglycerate
PEP	Phosphoenolpyruvate
Pyr	Pyruvate
Suc	Succinate
Mal	Malate
AMP	Adenosine monophosphate
ADP	Adenosine diphosphate
ATP	Adenosine triphosphate
GDP	Guanosine diphosphate
GTP	Guanosine triphosphate
CMP	Cytidine monophosphate
CDP	Cytidine diphosphate
CTP	Cytidine triphosphate
UMP	Uridine monophosphate
UDP	Uridine diphosphate
UTP	Uridine triphosphate
dAMP	Deoxy-adenosine monophosphate
dADP	Deoxy-adenosine diphosphate
dATP	Deoxy-adenosine triphosphate
dGDP	Deoxy-guanosine diphosphate
dGTP	Deoxy-guanosine triphosphate
dUMP	Deoxy-uridine monophosphate
dUTP	Deoxy-uridine triphosphate
dCTP	Deoxy-cytidine triphosphate
Gly	Glycine
Ala	Alanine
Ser	Serine
Pro	Proline
Val	Valine
Thr	Threonine
Ile	Isoleucine
Leu	Leucine
Asp	Aspartic acid
Gln	Glutamine
Glu	Glutamic acid
His	Histidine
Phe	Phenylalanine
Arg	Arginine
Lys	Lysine
